# A CRISPR/dCas9 toolkit for functional analysis of maize genes

**DOI:** 10.1186/s13007-020-00675-5

**Published:** 2020-10-02

**Authors:** Irene N. Gentzel, Chan Ho Park, Maria Bellizzi, Guiqing Xiao, Kiran R. Gadhave, Colin Murphree, Qin Yang, Jonathan LaMantia, Margaret G. Redinbaugh, Peter Balint-Kurti, Tim L. Sit, Guo-Liang Wang

**Affiliations:** 1 Department of Plant Pathology, The Ohio State University, 483B Kottman Hall, 2021 Coffey Road, Columbus, OH 43210 USA; 2 Department of Entomology and Plant Pathology, North Carolina State University, Raleigh, NC 27695 USA; 3 Corn, Soybean and Wheat Quality Research Unit, USDA-ARS, Wooster, OH 44691 USA

**Keywords:** Maize, Protoplasts, CRISPR/Cas9, Transcription activation, Transcription suppression

## Abstract

**Background:**

The Clustered Regularly Interspaced Short Palindromic Repeats (CRISPR)/Cas9 system has become a powerful tool for functional genomics in plants. The RNA-guided nuclease can be used to not only generate precise genomic mutations, but also to manipulate gene expression when present as a deactivated protein (dCas9).

**Results:**

In this study, we describe a vector toolkit for analyzing dCas9-mediated activation (CRISPRa) or inactivation (CRISPRi) of gene expression in maize protoplasts. An improved maize protoplast isolation and transfection method is presented, as well as a description of dCas9 vectors to enhance or repress maize gene expression.

**Conclusions:**

We anticipate that this maize protoplast toolkit will streamline the analysis of gRNA candidates and facilitate genetic studies of important trait genes in this transformation-recalcitrant plant.

## Background

The Clustered Regularly Interspaced Short Palindromic Repeats (CRISPR)/Cas9 system is the method of choice for plant genome editing projects, as it combines simplicity with efficiency and precision [1, 2]. It relies on the nuclease activity of the Cas9 protein, a component of adaptive bacterial defense against bacteriophages or other nucleic acid threats [3]. The specificity of this system is conferred by the interaction of the Cas9 nuclease with an RNA composed of a scaffold RNA joined to a guide RNA (gRNA), which directs the protein to a specific target DNA sequence for cleavage [4]. The site of mutagenesis can therefore be programmed simply by adjusting the sequence of the gRNA, provided a protospacer adjacent motif (PAM) sequence is present in the target DNA [4, 5]. The CRISPR/Cas9 system is also useful for other genetic manipulations beyond genomic sequence editing, such as regulation of gene expression and epigenetic modification [2, 4]. CRISPR-mediated transcription inhibition (CRISPRi) or activation (CRISPRa) is achieved by utilizing a nuclease-deactivated form of Cas9 (dCas9), a non-cutting variant which maintains its DNA-binding specificity [6, 7]. While the interaction of dCas9 itself with a specific promoter can reduce gene expression levels, fusion with a repression domain can enhance this effect [6]. One such repressor used in plant studies is the 12 amino acid SRDX domain, also known as an ERF-associated amphiphilic repression (EAR)-motif found in some transcriptional repressors [8, 9]. Conversely, dCas9 fused with an activation domain can be used to significantly elevate transcription from targeted native promoters [7, 10]. Recently, the dCas9-VP64 and dCas9-TV systems, which are based on modular repeats of the herpes simplex activation domain, were described as strong dCas9 activators of plant gene expression [10, 11].

The model species *Arabidopsis thaliana* can be easily transformed via the floral dip method with *Agrobacteria* delivery of genetic material, a process which can generate stable transgenic seed within 3–5 weeks [12]. In contrast, maize transformation methods are more laborious and time consuming, taking up to 6 months after the transformation event to generate transgenic seed [13–17]. Typically, immature embryo-derived callus tissue is transformed either by particle bombardment or *Agrobacterium*-mediated delivery of transgenes and both processes have a transformation rate efficiency of about 12–30% [13]. A further issue with maize transformation is the recalcitrance of most cultivars to callus production and plant regeneration, leading to the Hi-II hybrid as the most commonly used line [13, 16]. For many studies, however, it may be desirous to obtain transformants from other cultivars such as B73 or Mo17. Therefore, to produce transgenic plants with a high percentage of the desired parental background, it is necessary to perform 4–5 backcrosses, which adds another 1.5–2 years to the process [18–20]. Consequently, alternative techniques to quickly manipulate maize gene expression in the desired genetic background would be highly beneficial.

Protoplasts, live plant cells from which the cell wall has been removed, have proven to be a tractable system for a wide range of studies, including biochemistry, cell dedifferentiation, as well as genetic manipulation [21–24]. Methods for transient transgene expression in protoplasts are relatively quick and straightforward, making them a useful system when stable transgenic plants are unavailable or a high-throughput system is needed [24, 25]. It has been reported that electroporation and polyethylene glycol (PEG)-mediated transfection can be used to introduce plasmid DNA into maize protoplasts [26–28]. Therefore, in this study we describe our improvement of these published methods to generate high quality maize protoplasts suitable for gene expression analysis.

While online algorithms are available to aid in gRNA design, the list of top candidates provided nonetheless needs to be empirically verified for effective CRISPR/(d)Cas9 activity [29]. The CRISPRi and CRISPRa toolkits we describe offer a simple and time-efficient approach to facilitate screening gRNA candidates in maize protoplasts prior to the generation of stable transgenic lines, for example, which not only streamlines the gRNA selection process but also reduces the cost and time burdens associated with repeated maize transgenic production.

## Results

### Improvement of protoplast transfection conditions

**Fig. 1:**
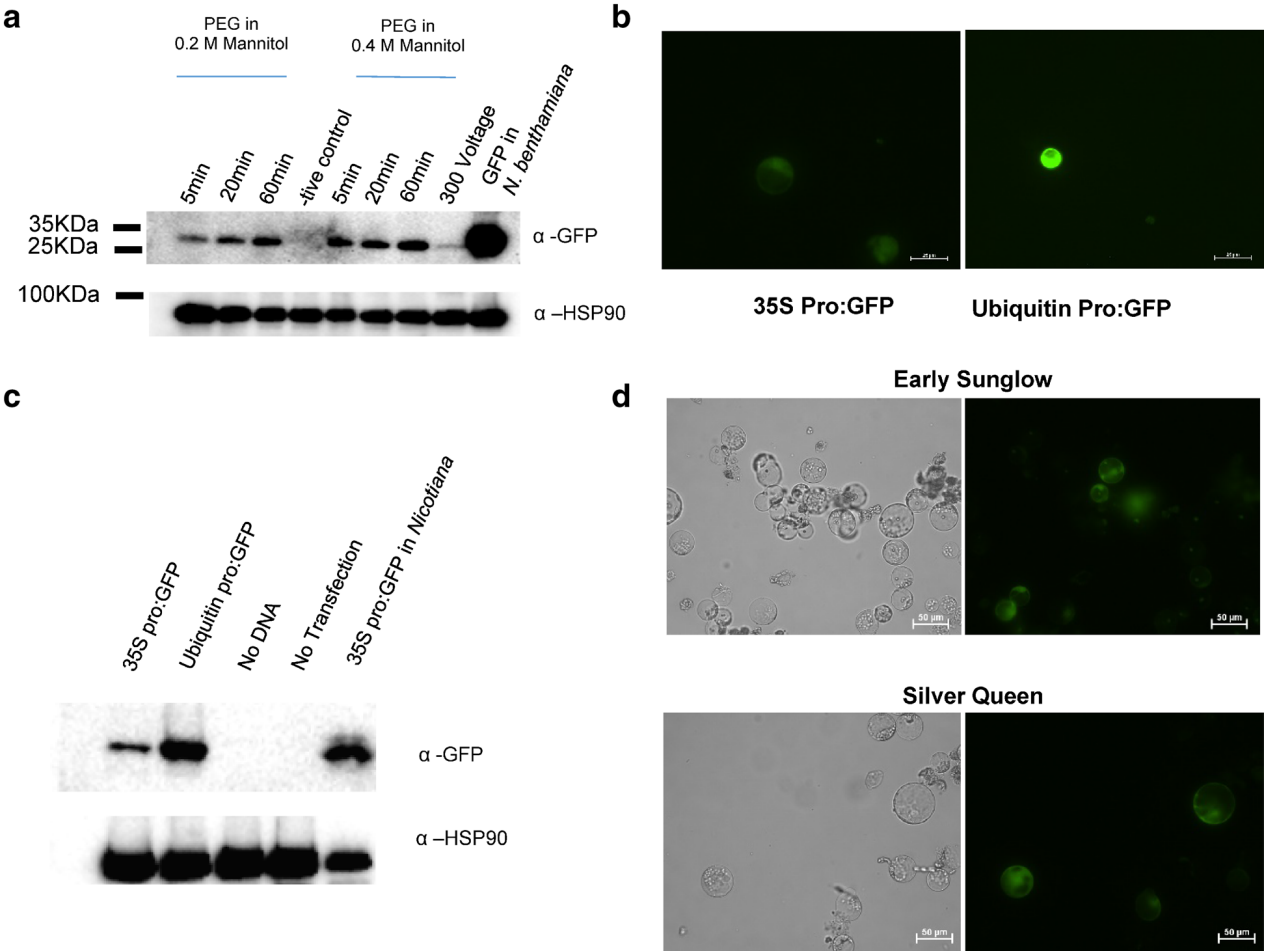
Analysis of protoplast transfection methods. **a** Western blot analysis of maize protoplasts transfected with pCXUN-HA-GFP under different mannitol concentrations and incubation length, in addition to electroporation at 300 V. GFP expression in *N. benthamiana* leaves is the positive control. **b** GFP expression driven by the 35S promoter (left) or the maize ubiquitin promoter (right) at 20× magnification. Exposure time was one second. **c** Western blot analysis of GFP expression in maize protoplasts driven by the 35S or ubiquitin promoters compared to the *N. benthamiana* positive control. **d** GFP expression in protoplasts from maize hybrids Early Sunglow (top) and Silver Queen (bottom) was observed at 20× magnification 4 days post transfection with pCXUN-HA-GFP. Images and blots are representative of two biological replicates

To develop a robust CRISPRa and CRISPRi system for maize protoplasts, we first analyzed the following protoplast transfection conditions. Protoplasts were isolated from 2-week-old etiolated maize seedlings as described in Burdo et al. [26]. Next, we compared electroporation with PEG-mediated transfection, where 40% PEG was prepared in either 0.2 M or 0.4 M mannitol as described for Arabidopsis and rice protoplast transfection, respectively [24, 30]. After transfection with a construct that carried a GFP-expression cassette (pCXUN-HA-GFP, Additional file 1), we conducted western blot analysis. As shown in Fig. 1a, maize protoplast transfection with 40% PEG in 0.4 M mannitol resulted in better expression of GFP compared to those with 0.2 M mannitol. As would be expected, an increase in incubation time resulted in higher GFP expression regardless of the concentration of mannitol.

Maize protoplasts were transfected with constructs carrying GFP driven by two promoters commonly used for high-level expression in maize: the cauliflower mosaic virus (CMV) 35S promoter or the maize ubiquitin promoter. According to microscopic observation (Fig. 1b) and western blot analysis (Fig. 1c), expression of GFP was stronger when driven by the maize ubiquitin promoter than that by the CMV 35S promoter. Further tests with protoplasts isolated from Early Sunglow and Silver Queen hybrids revealed their remarkable longevity after transfection with pCXUN-HA-GFP. As shown in Fig. 1d, GFP expression was detected in both hybrids 4 days post transfection (dpt). Based on these observations, we adopted 0.4 M mannitol for protoplast transfection and utilized the ubiquitin promoter in subsequent CRISPRa and CRISPRi vector construction. By comparing protoplasts with GFP signal to those without in a given area, we observed a transformation efficiency range of 60–70% which is similar to that reported for rice protoplasts [30].

### Expression of dCas9 variants *in planta*

**Fig. 2:**
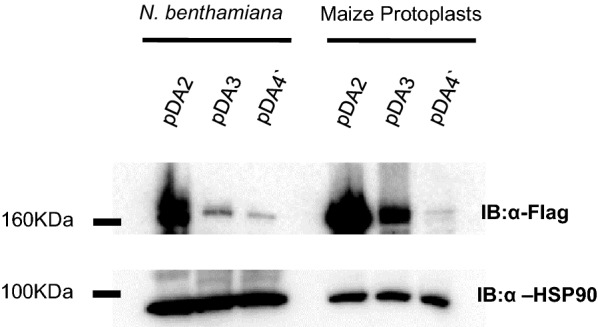
Expression of dCas9 variants *in planta*. Western blot expression analysis of pDA2 (dCas9), pDA3 (dCas9-VP64), pDA4 (dCas9-SRDX) constructs in *N. benthamiana* leaves (left) and maize protoplasts (right). Expression in these plant systems was assessed twice in separate experiments

To develop the maize CRISPRi and CRISPRa toolkit, we assembled the following series of constructs using a pTF101.1rev binary vector backbone: pDA2 (conferring dCas9 expression), pDA3 (dCas9-VP64, conferring dCas9-mediated expression activation), pDA4 (dCas9-SRDX, conferring dCas9-mediated expression repression), and pDA5 (dCas9-TV, conferring stronger dCas9-mediated expression activation). Each dCas9 derivative is N-terminally Flag-tagged and driven by the maize ubiquitin promoter (Additional file 1). Also included is a dual 35S-driven BAR cassette to confer glufosinate resistance. To confirm the expression of dCas9 variants *in planta* within whole plants, we infiltrated agrobacteria strains carrying different pDA vectors into *Nicotiana benthamiana* leaves. Three days after infiltration, total protein was isolated and expression of dCas9 variants was detected at the expected size using an anti-Flag antibody (Fig. 2). To confirm expression in maize plants by protoplast transient expression, we cloned the HindIII and SbfI fragment containing the dCas9 expression cassette from each pDA construct into the HindIII and PstI sites of pXUN, thereby decreasing the size of the construct by about 7 kb to increase the protoplast transfection rate. Total protein was isolated 16 h after maize protoplast transfection, and expression of the dCas9 variants were detected at the expected size by western blot with the anti-Flag antibody (Fig. 2). We observed that pDA2 showed higher expression compared to the pDA3 and pDA4 dCas9 variants in both *N. benthamiana* plants and maize protoplasts. While the reason is not clear, we speculate that the addition of activation and repression domains to the dCas9 construct leads to this expression reduction. Although a direct comparison to dCas9 was not included, Li et al. [10] observed a similar trend with western blot analysis of dCas9-activation constructs in *Arabidopsis* protoplasts, where increases in the overall size of the activation domains correlated with reduced expression.

### Testing the transcriptional changes of *ChlH* and *TrxH* using CRISPR/dCas9 constructs in protoplasts

**Fig. 3:**
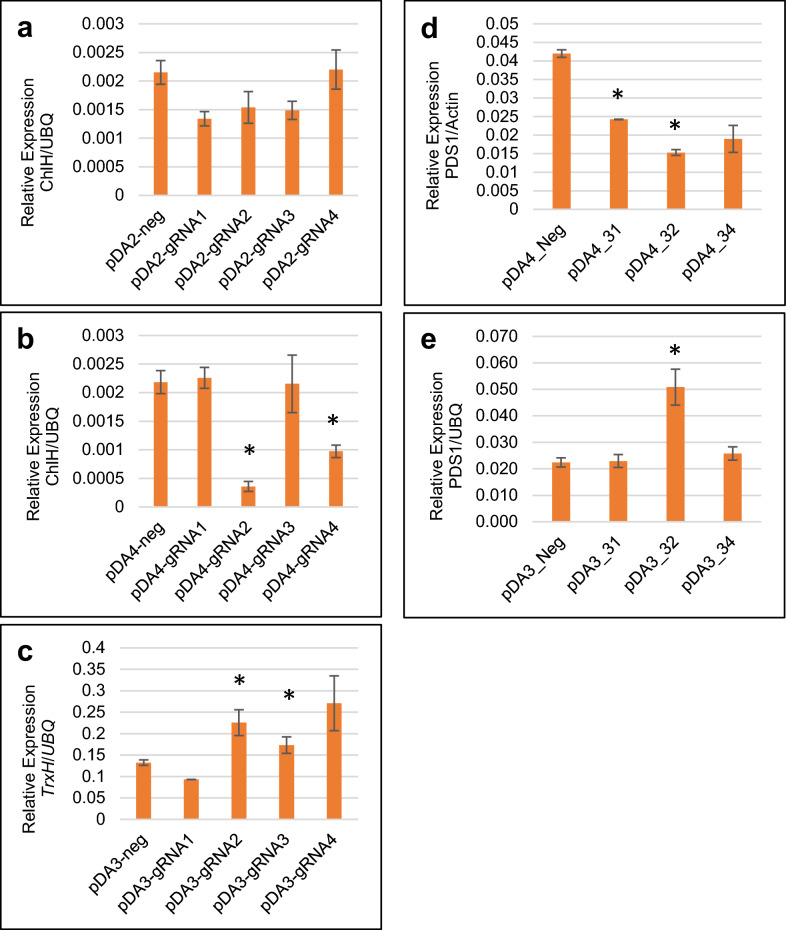
Target gene expression analysis in maize protoplasts transfected with CRISPRa and CRISPRi constructs. Maize protoplasts were co-transfected with pDA2 (**a**) or pDA4 (**b**) with individual *ChlH* gRNA candidates, or with pDA3 (**c**) and individual *TrxH* gRNA candidates. To test combinations of multiplexed *PDS1* gRNAs separated by tRNAs, maize protoplasts were co-transfected with pDA4 (**d**) or pDA3 (**e**). The ability of the gRNAs to repress (pDA2 and pDA4) or activate (pDA3) the target genes was assessed by qRT-PCR. Data represent triplicate samples from one biological replicate, where asterisks (*) indicate significant differences from the negative control, assessed by Student’s t-test where p < 0.1. Error bars are standard deviation

To determine the effectiveness of the pDA vectors for either CRISPRa or CRISPRi, we designed gRNAs targeting the promoter of the maize Subunit H of magnesium chelatase gene (*ChlH*), a marker gene whose mutation in whole plants leads to yellowing seedling phenotype due to defects in chloroplast development [31]. Four gRNAs targeting different regions of the *ChlH* promoter (Additional file 2) were designed and co-expressed with pDA2 (dCas9) or pDA4 (dCas9-SRDX) in protoplasts. qRT-PCR analysis showed that gRNA1, gRNA2, and gRNA3 co-transfection with pDA2 resulted in an approximate 25% reduction in *ChlH* expression, while gRNA4 had no effect (Fig. 3a). Co-expression of pDA4 with gRNA2 or gRNA4 resulted in nearly a 75% or 50% reduction in *ChlH* expression, respectively, compared to the negative control (Fig. 3b). These data show that while dCas9 has some transcription repression activity, this can be enhanced with the addition of the SRDX suppressor. For CRISPRa, we analyzed *Thioredoxin H* (*TrxH*), a gene whose increased transcription in whole plants confers resistance to sugarcane mosaic virus (SCMV) [32]. As with *ChlH*, we tested four gRNAs targeting the *TrxH* promoter (Additional file 2). When co-expressed with pDA3 (dCas9-VP64), gRNA2 or gRNA4 resulted in about a two-fold increase in *TrxH* transcripts (Fig. 3c). These two analyses confirmed that our pDA3 and pDA4 vectors can be used for CRISPRa and CRISPRi approaches, respectively, and gRNAs for target genes can be tested in maize protoplasts for further experiments.

### Testing the transcriptional changes of *PDS1* using three CRISPR/dCas9 constructs in maize protoplasts

To demonstrate CRISPRi with multiplexed constructs, gRNAs targeting the promoter of the maize *phytoene desaturase1* (*PDS1*) gene were designed. *PDS1* is a commonly used marker gene for virus-induced gene silencing (VIGS) as well as CRISPR/Cas9 genome editing analysis across a range of plant species, where silencing or mutation of the gene culminates in an easily observed photobleaching phenotype [33–37]. To test *PDS1* expression, we designed four gRNAs targeting the *PDS1* promoter (Additional file 2). Next, to multiplex two gRNAs in the same construct, we added three tRNAs to flank each side of the gRNA-scaffold sequences. The addition of tRNAs for multiplexed constructs is a widely-used method to create individual, discrete gRNAs in vivo from a single construct by the activity of the plant’s endogenous tRNA-processing machinery [38–40]. Multiple combinations of these four *PDS1* gRNAs were then co-transfected with either pDA2, pDA3, or pDA4 into maize protoplasts. We determined that a combination of gRNA2 and gRNA3 co-transfected with pDA4 showed a decrease of about 60% in *PDS1* transcription compared to the negative control, as measured by qRT-PCR (Fig. 3d). We also tested whether the *PDS1* gRNAs could be used for transcription activation with pDA3 (dCas9-VP64) in maize protoplasts. With pDA3, a combination with gRNA2 and gRNA3 showed about 2.5 times of *PDS1* transcription activation (Fig. 3e).

The dual-luciferase assay is rapid, sensitive, and reliable for analysis of transcriptional repressors, gene expression or functional interaction of signaling molecules [41]. The 35S-driven *Renilla* luciferase serves as an internal and transfection control, while Firefly luciferase is driven by a promoter of interest [42]. We tested whether this assay could be utilized to assess the repression or activation of *PDS1* using our CRISPR/dCas9 constructs. The vectors for this system were generated by first cloning a 1.4 kb promoter fragment of *PDS1* into the BamHI site of pGreenII-800-RNA1-Luc, resulting in the construct pGreenII-800-RNA1-*PDS1*:Luc (Additional file 3). Next, *PDS1* gRNAs were cloned into the BtgZI and BsaI sites with the different multiplex combinations as described above. After the co-transfection of *PDS1* gRNAs in pGreenII-800-RNA1-*PDS1*:Luc with CRISPR/dCas9 constructs, the protoplasts were lysed for Firefly and *Renilla* luciferase activity according to manufacturer’s instructions (Promega). As shown in the preliminary results of Additional file 3, the relative expression patterns of *PDS1* measured by the dual-luciferase assay were similar to that detected by qRT-PCR (Fig. 3d–e). Overall, a combination of gRNA2 and gRNA3 showed the best activation and suppression of *PDS1* with pDA3 and pDA4, respectively. As an additional control for the protoplast transfection rate, we modified the pGreenII-800-RNAi-Luc vector by inserting a GFP-expression cassette. Prior to gRNA analysis via dual-luciferase or qRT-PCR, the transfection rate with GreenII-800-RNAI-GFP-Luc can be determined by western blot analysis (Additional file 3).

## Discussion

### Development of the CRISPRa and CRISPRi toolkit for maize

In this study, we described an efficient system for testing gRNAs targeting trait genes for CRISPRa and CRISPRi in maize protoplasts. To develop this system, we first modified maize protoplast transfection methods to robustly express the dCas9-variants for CRISPRa and CRISPRi. Specifically, we utilized the isolation solution described by Burdo et al. [26] that had been developed for electroporation, and instead paired this with a modified cost-effective PEG-mediated transfection described by Cao et al. [27] and Yoo et al. [24]. Analysis of GFP-construct transfections revealed these modifications produced robust GFP expression (Fig. 1a–c). Additionally, we observed protoplasts remained viable and expressed GFP 4 days after transfection, indicating this protoplast isolation/transfection method would be suitable for multi-day experiments such as those requiring virus replication (Fig. 1d).

As with any CRISPR/(d)Cas9 system, it is important to empirically verify that the gRNA sequences predicted by design algorithms are indeed effective. Using our CRISPRa/CRISPRi protoplast system based on dCas9 (pDA2), dCas9-VP64 (pDA3) or dCas9-SRDX (pDA4) vectors, we tested the effectiveness of the top four gRNA candidates targeting maize promoters of *PDS1, ChlH,* or *TrxH,* respectively (Figs. 2 and 3)*.* Similar to other reports on dCas9 activity, we observed that pDA2 exhibited some suppression activity on *ChlH* expression (Fig. 3a), although the magnitude of this suppression was greater with pDA4 for some gRNAs (Fig. 3b). Tests with pDA3, the CRISPRa construct, revealed a two-fold increase in TrxH expression depending on which gRNA was co-transfected (Fig. 3c). The varying effectiveness between gRNAs observed in this study reiterates the necessity of testing multiple gRNA candidates.

In addition to testing single gRNA constructs, we also examined the effectiveness of multiplexed gRNAs on *PDS1* expression in protoplasts co-transfected with pDA3 or pDA4. We hypothesized that expressing more than one gRNA could increase the extent of transcription activation or repression, respectively. However, as the results in Fig. 3d–e revealed, the relative impact of multiple gRNAs on their target’s expression remained similar in magnitude to what we observed for single gRNAs in fig. 3a–c. Further research is needed to assess if both gRNAs within the multiplexed construct were contributing to the observed changes in *PDS1* expression.

### Application of toolkits for functional genomics

Our ultimate goal for this study was to produce the tools needed to modify agronomic traits such as disease resistance and abiotic stress tolerance by transiently changing the expression of multiple trait genes without extra transformation steps to deliver gRNAs specific to each target gene. In this study, we generated CRISPRa and CRISPRi vectors for maize protoplast transfection. Based on our results, we believe that this toolkit will be very useful for characterizing genes of interest in maize, as this approach has multiple advantages over conventional screening methods such as VIGS. Mainly, the CRISPRa/CRISPRi protoplast system provides a scalable means for convenient and cost effective analysis of both gene activation and suppression. With the protoplast system, it is possible to multiplex at least two gRNAs within the same vector and obtain CRISPR-mediated gene activation or suppression using the pDA3 and pDA4 vectors (Fig. 3). Ultimately, our new CRISPRa and CRISPRi toolkits will provide the maize community with useful materials for gene expression studies.

## Materials and methods

### Plant materials and growth conditions

Etiolated B73 maize (*Zea mays*) seedlings were prepared as follows. Kernels were imbibed for 24 h in room temperature water before being sown in moist ProMix (Sungro) soil. For 3 days, or until coleoptile emergence, plants were kept in a Conviron PGR15 growth chamber under a 12 h light (242 µmol)/12 h dark cycle, where temperatures and relative humidity were maintained at 26 °C/20 °C and 80%/60%, respectively. Plants were subsequently grown in complete darkness at 25 °C and 60% relative humidity for up to 2 weeks or until full expansion of the second true leaves.

### Maize protoplast isolation

Maize leaf protoplasts were prepared as described by Burdo et al. [26] with some modification. Briefly, etiolated B73 maize seedlings were cut into ~ 0.5 mm strips and placed in a flask containing 3% cellulase onozuka™ RS (Yakult Pharmaceutical Industry Co. Ltd., Japan), 0.7% macerozyme R10, 0.6 M mannitol, 10 mM KCl, 10 mM MES, 5 mM CaCl_2_, 0.1% (w/v) BSA. This enzyme solution was vacuum infiltrated into the leaf tissue for 30 min at 20 mmHg and followed by agitation at 40–50 rpm for 2.5–3 h. To release the protoplasts, the flask was shaken at 90 rpm for 30 min. Protoplasts were filtered out of the enzyme solution through a 35 µm nylon mesh and collected by centrifugation at 300×*g* for 2 min. The protoplast pellet was washed twice with W5 media (154 mM NaCl, 125 mM CaCl_2_, 5 mM KCl, 5 mM glucose, and 2 mM MES) prior to resuspension in MMG buffer (0.4 M mannitol, 15 mM MgCl_2_ and 4 mM MES) for DNA transfection.

### Protoplast transfection

Protoplast transfection was conducted based on the method described by Chen et al. [30] with a few modifications. Briefly, 100 µL of protoplasts resuspended in MMG buffer at a density of 1–1.5 × 10^6^ protoplasts per mL were mixed with 10 µg of each plasmid followed by the addition of 110 µL freshly prepared 40% PEG-CaCl_2_ solution (40% PEG-4000, 0.4 M mannitol and 0.1 M CaCl_2_) and incubation for 5 min to 1 h at room temperature for Fig. 1a analysis. For subsequent experiments, the 1 h incubation step was used. After incubation, the protoplasts were washed with 1 mL of W5 buffer to remove PEG. After centrifugation at 300×*g* for 3 min, the protoplasts were resuspended in 1 mL of W5 buffer and incubated for 16 h at room temperature.

### Dual-luciferase assay

Protoplasts incubated for 16 h in darkness at room temperature were harvested by centrifugation at 300 × g for 3 min. The luciferase assay was conducted according to the manufacturer’s instructions (Promega). Briefly, 50 µL of 1X Passive lysis buffer was added to the harvested protoplasts and mixed by pipetting 4–5 times. After centrifugation at 12,000 × g for 5 min, 10 µL of supernatant was mixed with 40 µL of LARII buffer and firefly luciferase activity was measured for 1 min using a Glomax 20/20 luminometer. Next, 40 µL of Stop&Glo solution was added to the sample to measure *Renilla* luciferase activity as an internal control. Three technical replicates were conducted for each sample.

### Vector construction

pDA2-5 vectors were developed using pTF101.1.rev (kindly provided by Kan Wang, Iowa State University) as a backbone. Other components were cloned from vectors previously published; the ubiquitin promoter and nos terminator were cloned from pXUN [43], dCas9-VP64 and dCas-SRDX were cloned from pYPQ152 or pYPQ153, respectively [8], and dCas9-TV was cloned from pCambia-dCas9-TV [10]. Cloning was conducted using traditional restriction enzyme digestion and ligation followed by PCR screening and Sanger sequencing confirmation. For protoplast transient expression vectors, we cloned the HindIII and SbfI fragment from each pDA# construct into the HindIII and PstI sites of pXUN, which decreases the size of the construct but increases efficiency of protoplast transfection. To clone gRNAs in the expression vector, PstI and SalI fragment containing the gRNA expression cassette from pENTR-gRNA1 was cloned to the pGreenII-800-Luc vector [42], which was designated as pGreenII-800-RNA1-Luc.

### Cloning of gRNAs in pGreenII-800-Luc vector

Four gRNAs targeting promoter regions of maize *PDS1, ChlH,* and *TrxH* were designed using the CRISPR-P website (https://crispr.hzau.edu.cn/CRISPR2/), which was based on the B73 (AGPv.3.21) reference genome at the time the analysis was completed. *PDS1* gRNA3 was cloned in the BtgZI site and gRNA1, gRNA2 and gRNA4 were cloned in the BsaI site, resulting in two gRNAs per construct. Each of the four *ChlH* and *TrxH* gRNAs was tested individually.

### Western blot

Total protein from frozen plant tissue was isolated from mortar-ground leaves using a 100 mg tissue/400 µL ratio of extraction buffer (150 mM NaCl, 20 mM Tris, pH7.5, 1 mM EDTA, 1% Triton X-100, 0.1% SDS, 1X protease inhibitor cocktail, and 5 µl/mL 1 M DTT) as described in Geng et al. [44]. Following a 5 min incubation on ice, the extracts were centrifuged at 16,000 × g for 10 min at 4 °C. Supernatants were mixed with SDS loading dye and boiled for 5 min before being loaded on a 7.5% SDS-PAGE gel. Following membrane transfer, total protein was assessed with Ponceau staining. dCas9 proteins were detected with an anti-flag antibody and α-HSP90 was used as the loading control.

## Supplementary information

**Additional file 1.** Vector Maps. (A) pCXUN-HA-GFP used for testing protoplast transfection conditions. (B) Diagram of each pDA vector showing an ubiquitin-driven, Flag-tagged dCas9 with IV2 intron followed by transcription activators (VP64 and TAL-VP128) or suppressor (SRDX). A dual 35S promoter drives BAR for glufosinate resistance.

**Additional file 2.** gRNA sequences used in this study.

**Additional file 3.** Dual luciferase assay components and preliminary results. (A) pGreenII-800-RNAI-PDS1-Luc dual luciferase construct, where Renilla luciferase provides an internal control for PDS1-driven Firefly luciferase. Dual luciferase assay using maize protoplasts co-transfected with indicated PDS1 gRNAs and dCas9-SRDX (B) or dCas9-VP64 (C). Data shown in B and C are from one biological replicate. An additional control construct was developed where GFP was expressed from the dual luciferase vector pGreenII-800-RNAI-Luc (D). Expression was detected by Western blot (E), which shows four separate maize protoplast transfection samples with this construct, compared to the no transfection, no DNA, and positive controls.

## Data Availability

The datasets used and/or analyzed during the current study are available from the corresponding author on reasonable request.

## References

[1] Zhang Y, Xie X, Liu YG, Zhang Y, Xie X, Liu YG, et al. CRISPR/Cas9-Based Genome Editing in Plants. 1st ed. Prog. Mol. Biol. Transl. Sci. Elsevier Inc.; 2017. 10.1016/bs.pmbts.2017.03.008 28712494

[2] Moradpour M (2019). Abdulah SNA CRISPR/dCas9 platforms in plants: strategies and applications beyond genome editing. Plant Biotechnol J..

[3] Hryhorowicz M, Lipiński D, Zeyland J, Słomski R (2017). CRISPR/Cas9 immune system as a tool for genome engineering. Arch Immunol Ther Exp (Warsz).

[4] Wang H, La Russa M, Qi LS (2016). CRISPR/Cas9 in genome editing and beyond. Annu Rev Biochem.

[5] Filippova J, Matveeva A, Zhuravlev E, Stepanov G (2019). Guide RNA modification as a way to improve CRISPR/Cas9-based genome-editing systems. Biochimie.

[6] Qi LS, Larson MH, Gilbert LA, Doudna JA, Weissman JS, Arkin AP (2013). Repurposing CRISPR as an RNA-guided platform for sequence-specific control of gene expression. Cell.

[7] Gilbert LA, Larson MH, Morsut L, Liu Z, Brar GA, Torres SE (2013). CRISPR-mediated modular RNA-guided regulation of transcription in eukaryotes. Cell..

[8] Lowder LG, Zhang D, Baltes NJ, Paul JW, Tang X, Zheng X (2015). A CRISPR/Cas9 toolbox for multiplexed plant genome editing and transcriptional regulation. Plant Physiol.

[9] Hiratsu K, Matsui K, Koyama T, Ohme-Takagi M (2003). Dominant repression of target genes by chimeric repressors that include the EAR motif, a repression domain, in Arabidopsis. Plant J.

[10] Li Z, Zhang D, Xiong X, Yan B, Xie W, Sheen J (2017). A potent Cas9-derived gene activator for plant and mammalian cells. Nat Plants Palgrave Macmillan Ltd.

[11] Beerli RR, Segal DJ, Dreier B, Iii CFB (1998). Toward controlling gene expression at will: Specific regulation of the *erbB-2/HER-2* promoter by using polydactyl zinc finger proteins constructed from modular building blocks. Proc Natl Acad Sci..

[12] Zhang X, Henriques R, Lin SS, Niu QW, Chua NH (2006). Agrobacterium-mediated transformation of *Arabidopsis thaliana* using the floral dip method. Nat Protoc.

[13] Yadava P, Abhishek A, Singh R, Singh I, Kaul T, Pattanayak A (2017). Advances in maize transformation technologies and development of transgenic maize. Front Plant Sci.

[14] Altpeter F, Springer NM, Bartley LE, Blechl AE, Brutnell TP, Citovsky V (2016). Advancing crop transformation in the era of genome editing. Plant Cell.

[15] Clough SJ, Bent AF (1998). Floral dip: a simplified method for Agrobacterium-mediated transformation of *Arabidopsis thaliana*. Plant J.

[16] Horn ME, Harkey RL, Vinas AK, Drees CF, Barker DK, Lane JR (2006). Use of Hi II-elite inbred hybrids in Agrobacterium-based transformation of maize. Vitr Cell Dev Biol - Plant.

[17] AU - Masters A, AU - Kang M, AU - McCaw M, AU - Zobrist JD, AU - Gordon-Kamm W, AU - Jones T, et al. Agrobacterium-mediated immature embryo transformation of recalcitrant maize inbred lines using morphogenic genes. JoVE. 2020. Doi: 10.3791/60782 . 10.3791/60782 32116304

[18] Baltes NJ, Gil-Humanes J, Voytas DF. Prog Mol Biol Transl Sci. 1st ed. Amsterdam: Elsevier Inc; 2017. 10.1016/bs.pmbts.2017.03.011 PMC8409219 28712492

[19] Young ARJ, Narita M, Narita M (2018). Maize methods and protocols.

[20] Falk DE (2010). Generating and maintaining diversity at the elite level in crop breeding. Genome.

[21] Jiang F, Zhu J, Liu HL (2013). Protoplasts: a useful research system for plant cell biology, especially dedifferentiation. Protoplasma.

[22] Hall RD (1988). Developments in plant protoplast research. Trends Biotechnol.

[23] Bajaj YPS (1974). Potentials of protoplast culture work in agriculture. Euphytica.

[24] Yoo SD, Cho YH, Sheen J (2007). Arabidopsis mesophyll protoplasts: a versatile cell system for transient gene expression analysis. Nat Protoc.

[25] Page MT, Parry MAJ, Carmo-Silva E (2019). A high-throughput transient expression system for rice. Plant Cell Environ.

[26] Burdo B, Gray J, Goetting-Minesky MP, Wittler B, Hunt M, Li T (2014). The Maize TFome - development of a transcription factor open reading frame collection for functional genomics. Plant J Wiley.

[27] Cao J, Yao D, Lin F, Jiang M (2014). PEG-mediated transient gene expression and silencing system in maize mesophyll protoplasts: a valuable tool for signal transduction study in maize. Acta Physiol Plant.

[28] Gao L, Shen G, Zhang L, Qi J, Zhang C, Ma C (2019). An efficient system composed of maize protoplast transfection and HPLC-MS for studying the biosynthesis and regulation of maize benzoxazinoids. Plant Methods BioMed Central.

[29] Lee CM, Davis TH, Bao G (2018). Examination of CRISPR/Cas9 design tools and the effect of target site accessibility on Cas9 activity. Exp Physiol.

[30] Chen S, Tao L, Zeng L, Vega-Sanchez ME, Umemura K, Wang G-L (2006). A highly efficient transient protoplast system for analyzing defence gene expression and protein-protein interactions in rice. Mol Plant Pathol.

[31] Zoschke R, Chotewutmontri P, Barkan A (2017). Translation and co-translational membrane engagement of plastid-encoded chlorophyll-binding proteins are not influenced by chlorophyll availability in maize. Front Plant Sci.

[32] Liu Q, Liu H, Gong Y, Tao Y, Jiang L, Zuo W, Yang Q, Ye J, Lai J, Wu J, Lübberstedt T, Xu M (2017). An atypical thioredoxin imparts early resistance to sugarcane mosaic virus in maize. Molecular Plant.

[33] Singh DK, Lee HK, Dweikat I, Mysore KS (2018). An efficient and improved method for virus-induced gene silencing in sorghum. BMC Plant Biol.

[34] Mei Y, Zhang C, Kernodle BM, Hill JH, Whitham SA (2016). A Foxtail mosaic virus vector for virus-induced gene silencing in maize. Plant Physiol.

[35] Jarugula S, Willie K, Stewart LR (2018). Barley stripe mosaic virus (BSMV) as a virus-induced gene silencing vector in maize seedlings. Virus Genes.

[36] Kaur N, Alok A, Shivani, Kaur N, Pandey P, Awasthi P, et al. CRISPR/Cas9-mediated efficient editing in phytoene desaturase (PDS) demonstrates precise manipulation in banana cv. Rasthali genome. Funct Integr Genomics. 2018;18:89–99. 10.1007/s10142-017-0577-5 29188477

[37] Charrier A, Vergne E, Dousset N, Richer A, Petiteau A, Chevreau E (2019). Efficient targeted mutagenesis in apple and first time edition of pear using the CRISPR-Cas9 system. Front Plant Sci..

[38] Xie K, Minkenberg B, Yang Y (2015). Boosting CRISPR/Cas9 multiplex editing capability with the endogenous tRNA-processing system. Proc Natl Acad Sci..

[39] Mikami M, Toki S, Endo M (2017). In planta processing of the SpCas9-gRNA complex. Plant Cell Physiol.

[40] Ellison EE, Nagalakshmi U, Gamo ME, Huang P jui, Dinesh-Kumar S, Voytas DF. Multiplexed heritable gene editing using RNA viruses and mobile single guide RNAs. Nat Plants. Springer US; 2020; 10.1038/s41477-020-0670-y 32483329

[41] Wehner N, Hartmann L, Ehlert A, Böttner S, Oñate-Sánchez L, Dröge-Laser W (2011). High-throughput protoplast transactivation (PTA) system for the analysis of Arabidopsis transcription factor function. Plant J.

[42] Hellens RP, Allan AC, Friel EN, Bolitho K, Grafton K, Templeton MD (2005). Transient expression vectors for functional genomics, quantification of promoter activity and RNA silencing in plants. Plant Methods.

[43] Chen S, Songkumarn P, Liu J, Wang GL (2009). A versatile zero background T-vector system for gene cloning and functional genomics. Plant Physiol.

[44] Geng X, Shen M, Kim JH, Mackey D (2016). The Pseudomonas syringae type III effectors AvrRpm1 and AvrRpt2 promote virulence dependent on the F-box protein COI1. Plant Cell Rep..

