# Supplementary figures and images for: A CRISPR/dCas9 toolkit for functional analysis of maize genes
Source: Plant Methods. 2020 Oct 2;16:133. doi: 10.1186/s13007-020-00675-5 (PMC7532566; doi:10.1186/s13007-020-00675-5)

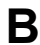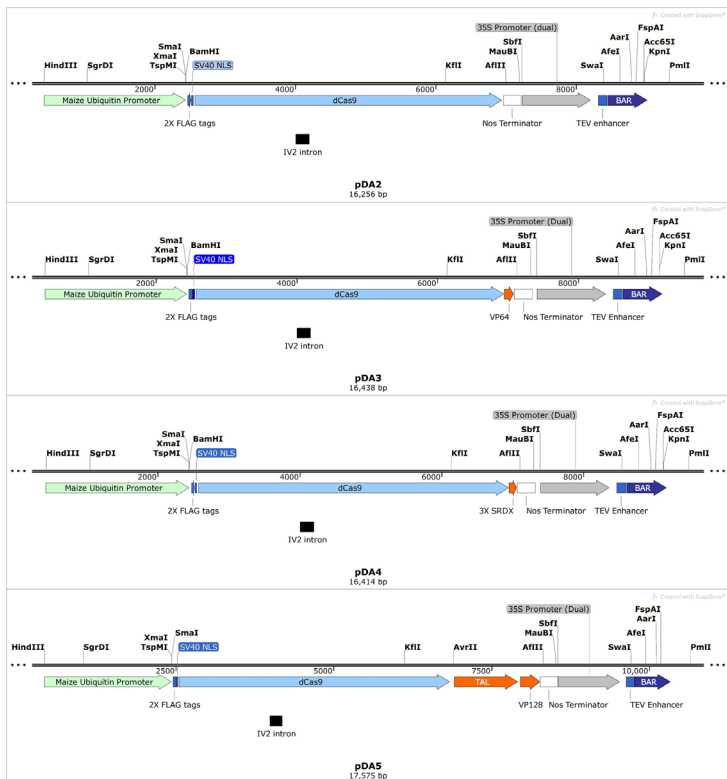

Supplement: Supplementary file 1 — Additional file 1. Vector Maps. (A) pCXUN-HA-GFP used for testing protoplast transfection conditions. (B) Diagram of each pDA vector showing an ubiquitin-driven, Flag-tagged dCas9 with IV2 intron followed by transcription activators (VP64 and TAL-VP128) or suppressor (SRDX). A dual 35S promoter drives BAR for glufosinate resistance. [file 13007_2020_675_MOESM1_ESM.pdf]

# A

Created with SnapGene®

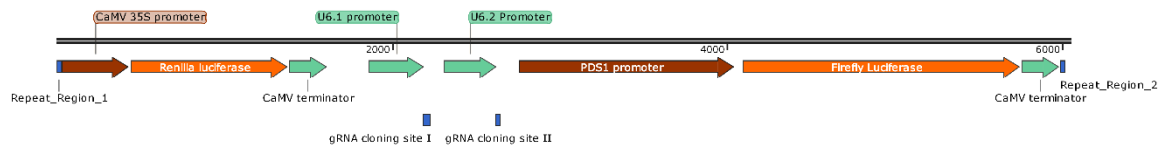

# B

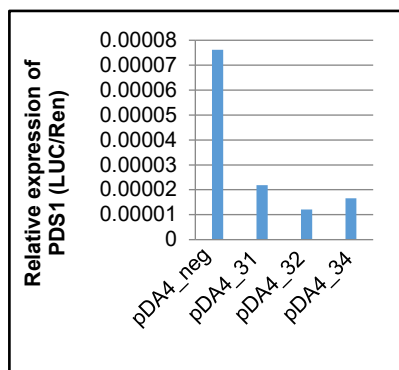

# C

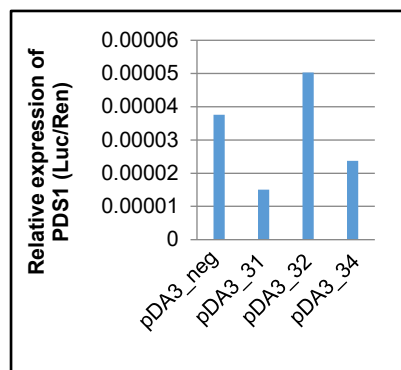

# D

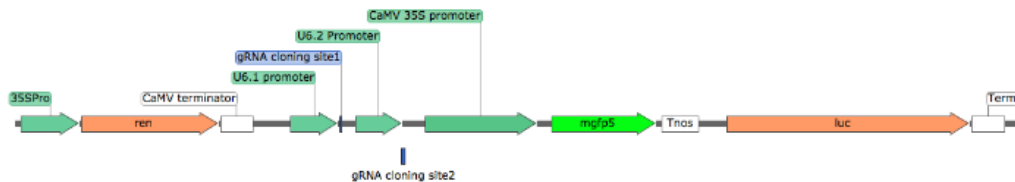

# E

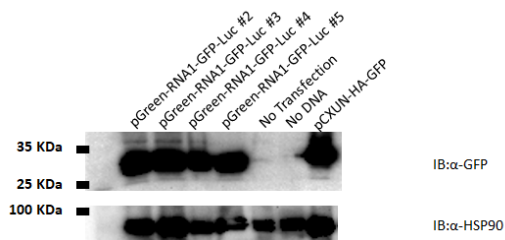

Supplement: Supplementary file 3 — Additional file 3. Dual luciferase assay components and preliminary results. (A) pGreenII-800-RNAI-PDS1-Luc dual luciferase construct, where Renilla luciferase provides an internal control for PDS1-driven Firefly luciferase. Dual luciferase assay using maize protoplasts co-transfected with indicated PDS1 gRNAs and dCas9-SRDX (B) or dCas9-VP64 (C). Data shown in B and C are from one biological replicate. An additional control construct was developed where GFP was expressed from the dual luciferase vector pGreenII-800-RNAI-Luc (D). Expression was detected by Western blot (E), which shows four separate maize protoplast transfection samples with this construct, compared to the no transfection, no DNA, and positive controls. [file 13007_2020_675_MOESM3_ESM.pdf]
