# Supplementary material for: A CRISPR/dCas9 toolkit for functional analysis of maize genes
Source: Plant Methods. 2020 Oct 2;16:133. doi: 10.1186/s13007-020-00675-5 (PMC7532566; doi:10.1186/s13007-020-00675-5)
Supplement: Supplementary file 2 — Additional file 2. gRNA sequences used in this study. [file 13007_2020_675_MOESM2_ESM.pdf]

Additional File 1. gRNA sequences used in this study

Maize gRNA targets	5' – 3' sequence	
PDS1 promoter	gRNA1	tctcgtcactatgtattccg
	gRNA2	cgggttagcggtatatacca
	gRNA3	tgtattccgcggcatcacac
	gRNA4	tctgttgctaaggtaatatg
ChlH promoter	gRNA1	acactattataattatgcaa
	gRNA2	gatcaatcgcacagttaggg
	gRNA3	ccaagcctctgccctcgacg
	gRNA4	tctcctcctccaggagacga
TrxH promoter	gRNA1	tgtcgtcccagcctgaaaaa
	gRNA2	gtacgtacaagctcacaaag
	gRNA3	cagctaggtgaatgcaacca
	gRNA4	gcggatttatgtctcggtag
**gRNA scaffold**	gttttagagctagaaatagcaagttaaaataaggctagtccgttatcaacttgaaaaagtggcaccgagtcggtgc	
**tRNA**	aacaaagcaccagtggtctagtggtagaatagtaccctgccacggtacagacccgggttcgattcccggctggtgca	
